# Phage Display-based Strategies for Cloning and Optimization of Monoclonal Antibodies Directed against Human Pathogens

**DOI:** 10.3390/ijms13078273

**Published:** 2012-07-04

**Authors:** Nicola Clementi, Nicasio Mancini, Laura Solforosi, Matteo Castelli, Massimo Clementi, Roberto Burioni

**Affiliations:** Microbiology and Virology Unit, “Vita-Salute” San Raffaele University, Milan 20132, Italy; E-Mails: mancini.nicasio@hsr.it (N.M.); solforosi.laura@hsr.it (L.S.); clementi.massimo@hsr.it (M.C.); m.castelli@studenti.unisr.it (M.C.); burioni.roberto@hsr.it (R.B.)

**Keywords:** phage display, library construction, monoclonal antibodies, biopanning, hypervariable pathogens

## Abstract

In the last two decades, several phage display-selected monoclonal antibodies (mAbs) have been described in the literature and a few of them have managed to reach the clinics. Among these, the anti-respiratory syncytial virus (RSV) Palivizumab, a phage-display optimized mAb, is the only marketed mAb directed against microbial pathogens. Palivizumab is a clear example of the importance of choosing the most appropriate strategy when selecting or optimizing an anti-infectious mAb. From this perspective, the extreme versatility of phage-display technology makes it a useful tool when setting up different strategies for the selection of mAbs directed against human pathogens, especially when their possible clinical use is considered. In this paper, we review the principal phage display strategies used to select anti-infectious mAbs, with particular attention focused on those used against hypervariable pathogens, such as HCV and influenza viruses.

## 1. Introduction

The basis of phage display technology is based on the presentation of peptides or protein fragments on the surface of bacteriophages (“phages”). It was first introduced to investigate the affinity enrichment of fusion proteins encoded by specific cDNA sequences included in the phage particles [1]. This is obtained by fusing the genes encoding the different peptides to the gene encoding a phage structural protein. The phage protein fused to the peptide represents an anchor for the displayed peptide and ideally should not interfere with the subsequent peptide-ligand interaction. The extreme versatility of the technique itself, subsequently improved on through the introduction of multiple peptide libraries and affinity selection procedures (“biopanning”) [2], was of immediate interest to the scientific community because of the potential variety of its uses. In particular, the possibility to construct more complex protein libraries, such as antibody fragments libraries [3], opened new perspectives for the specific targeting of human microbial pathogens by monoclonal antibodies (mAbs) obtained through affinity selection strategies. Since the last century, the therapeutic potential of mAbs against human pathogens has been regularly reported. It has also been described how mAbs directed against conserved regions could represent a new tool for the improvement of available therapeutic or prophylactic strategies against hypervariable pathogens. In this review, we provide an overview of the most promising phage display strategies used in the cloning of anti-infectious mAbs, reporting for each strategy specific examples of available mAbs. Phage display antibody library selection largely depends upon various molecular factors such as methods for phage library construction, phage/phagemid vectors, host cells and biopanning process. However, in this review particular attention is focused on the different strategies employed for the selection of rare mAbs directed against conserved epitopes of hypervariable pathogens, such as hepatitis C virus (HCV) and influenza viruses, and avoids technical details explaining the principal steps of the phage display method.

## 2. Phage Display: The Importance of the Correct Strategy

**Figure 1:**
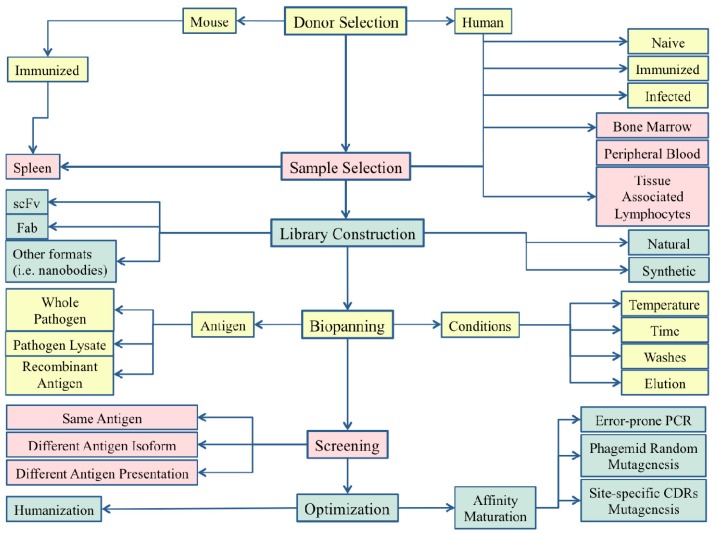
Crossroads representing the different decisional schemes used for the successful monoclonal antibodies (mAbs) molecular cloning described in the paper.

Phage display technology is an extremely versatile technique whose final success may depend on the choices made during the set-up of a cloning strategy (Figure 1). Generally speaking, there are three main choices that need to be made depending on the intended future use of the selected mAbs: (i) the type of library to be constructed and the original source of B cells to be used; (ii) the selection strategy employed in the biopanning procedure; and (iii) the possible need for post-selection phage display-based mAb optimization. These three points will now be addressed with specific examples of available phage display-derived mAbs. As already stated, particular attention will be paid to successful phage display strategies that have been used for the selection of broad-range mAbs directed against hypervariable human pathogens, such as HCV and influenza viruses.

### 2.1. The Library Design and the Source of B-Cells

The main advantage of the phagemidic system is that the protein displayed on the surface of the phage is encoded by the DNA enclosed in the phagemidic DNA. Several phagemid vectors have been described for this purpose in which the coat proteins of a filamentous phage (M13) can be fused to foreign proteins. Currently, the most commonly utilized vectors use the minor coat protein (pIII) or the major coat protein (pVIII) for such fusion. The careful construction of a phage display antibody fragments library is, therefore, the most important step for the success of mAb cloning. The first important choice is the selection of the source of B-cells to use for mRNA extraction and the subsequent amplification of the antibody variable genes to be cloned into the vectors. To date, there have been no significant studies comparing different B-cell sources in differing conditions [4]. In the main, libraries have been constructed successfully from human bone marrow [5–8], peripheral blood lymphocytes [9–11] and peripheral lymphoid tissues [12–17], as well as from murine spleen, peripheral B cells or from other animals such as sharks [18], chickens [18] and camelids [19,20]. The choice as to which samples to use should be based on the final objective of the research. For example, in order to clone mAbs to be used in clinical practice, human B-cells are the most appropriate sample; otherwise, antibodies to be used in diagnostics could be more easily obtained from murine B-cells derived from immunized mice. Moreover, an important decision is required through the choice of the mAb format to use. The molecules displayed on the phage surface are usually either monovalent Fab fragments or single chain Fv fragments (scFv). Fabs are constituted of two chains: the heavy chain Fd (constituted by the VH and CH1 domains) and the whole light chain, whereas scFv_s_ consist of a single protein containing the variable regions of both heavy and light chains (VH and VL) fused by a flexible aminoacidic linker. The choice of the antibody format also depends on the researcher’s purpose since Fabs and scFv_s_ often feature different affinity and pharmacokinetic properties. Another crucial variable in the library design is in the choice of the antibody isotype or of the discrete antibody subfamilies to be cloned.

These observations are simply the “tip of the iceberg” amongst all the possible options presenting when setting up a strategy for mAb molecular cloning (Table 1). That said, three specific examples depicting three possible different approaches are reported below: the first using a fully synthetic library for the selection of anti-*Clostridium botulinum* neurotoxin mAbs, and the other two using libraries from human bone marrow or peripheral B cells for the selection of anti-influenza A mAbs.

#### 2.1.1. Fully Synthetic Library Design

Antibody libraries can be obtained either from cDNA antibody sequences derived from the B cells of animal or human origin, or synthetically generated using random nucleotide sequences within selected CDRs in combination with one or multiple framework regions to replicate the diversity of a natural antibody repertoire [21]. These sequences are then fused to the sequence encoding the gene III phage coat protein allowing the display of the antibody fragment [22]. The construction of a fully synthetic Ab library has certain advantages particularly in cases such as the production of mAbs against highly lethal toxins, since the use of animals may be troublesome due to the toxic effects on the immunized animal. Another potential advantage in the use of a fully synthetic library is the possibility of enriching it in “antigen-specific” or rare V gene subfamilies in order to increase the probability of selecting mAb with the desired specificity [23]. We report as an example the construction of a fully synthetic library for the selection of antibodies capable of binding *Clostridium botulinum* neurotoxins serotype A (BoNT/A). BoNTs are the most lethal proteins known and are grouped in seven serotypes (A–G). A fully synthetic human scFv phage display library (1.35 × 10^10^ total number of clones) was constructed using VH3 and VH5 genes as master frameworks for the heavy chains (HC), and Vκ1, Vκ3, Vλ1 and Vλ3 genes as master frameworks for the light chains. The choice was made according to their high frequency in the human antibody repertoire, analyzing the statistical distributions of human CDR3s VH and VL belonging to differently described antibodies available in on-line specific databases [24,25]. The library was then screened against BoNT/A, decreasing the antigen concentration at each selection round. After panning selection, six different BoNT/A-specific scFv clones were selected and characterized by DNA sequencing. Although the library contained Vλ and Vκ light chain genes, as well as VH3, VH4, and VH5 heavy chain genes, all VL genes of the selected clones belonged to the Vλ3 gene family, whereas all VH genes belonged to the VH5 gene family except for one belonging to the VH3 gene family [23]. This example, demonstrates the potential benefits of synthetic libraries, which may be used when it is not possible to have access to materials from infected or vaccinated humans or animals. However, it is also important to keep in mind the potential drawbacks of such an approach. In particular, the initial choice of using discrete antibody subfamilies inevitably introduces a bias that could hamper the final results. Moreover it has been demonstrated that synthetic libraries may feature a high frequency of unnatural amber stop codons and glycosylation sites which can limit the conversion of the selected clones into IgG [22]. The possible loss of specificity of scFv_s_ selected from synthetic libraries when converted into whole IgG has also been described [22].

#### 2.1.2. Human Libraries from Bone Marrow and Peripheral Blood B-Cells

The importance of proper donor selection and of a correct B-cell source is closely related to the cloning purpose. In fact, even if mAbs derived from animal models can be optimized for the administration in human therapy or prophylaxis, a fully human mAb is certainly preferred. From this perspective, two examples regarding the molecular cloning of broadly neutralizing human mAbs directed against influenza A viruses are provided. As evidenced in the second part of the paragraph below, both approaches share the use of human B cells whose origin is however different.

Influenza virus A is one of the most variable human pathogens. It is important to try to identify and eventually elicit a broad-range immunity directed against broadly conserved viral regions [26–30]. Many approaches have been proposed in the literature [31–37], but a central role (in the prophylactic as well as in the therapeutic field) may be played by broad-range neutralizing human monoclonal antibodies (mAbs) throughout the identification of human B epitopes widely shared among different influenza subtypes [31,38]. It is known that antibodies are important in natural protection against influenza viruses, and that hemagglutinin (HA) represents the major target of the virus-neutralizing antibody response [39,40]. However, although a single influenza infection provides immunity against the same infecting virus and a limited number of related strains, the human host still remains susceptible to infection of antigenically drifted variants. This is due to the high variability of influenza viruses, mainly involving HA, but also possibly from the “original antigenic sin” phenomenon, according to which there is a lack of activation of naïve B cells recognizing novel protective epitopes, since Abs are mainly produced against epitopes of previously encountered viral strains. We report two examples showing different approaches for Ab-phage display library construction.

The first strategy was based on the construction of a library from bone marrow samples of highly pathogenic H5N1 influenza survivors [41]. The bone marrow is particularly rich in B cells, and its use in the construction of an antibody library allows the broadest variability. In the reported example, each λ and κ light chain family was amplified using family-specific VL and random JL primers. Regarding the heavy chain families, VH1, 3, 4 and 7 were recovered individually, while VH2, 5 and 6 were pooled. Each VL family was then separately cloned into the phagemid display vector in order to eliminate the bias among different families; subsequently, each VH family was cloned into the vector already containing the light chains. Antibodies were then exposed on the phage surface fused with pIII phage protein. Both scFv and Fab libraries were constructed.

Another possibility is the construction of an antibody library from peripheral blood B-lymphocytes. The peripheral blood may be enriched in antigen-specific B cell populations a few days after contact with a given pathogen. Conversely from the use of bone marrow-derived cells, it is important to choose convalescent or recently vaccinated donors [42]. We report the example of the cloning strategy of other broad-range anti-HA mAbs derived from healthy donors vaccinated with seasonal anti-influenza vaccine [43]. In this case, IgM memory B-cells from ten healthy donors were sorted from peripheral blood samples. After RNA extraction and retrotranscription, IgM-specific primers were used to ensure IgM VH repertoire selective amplification. Each individual scFv library was finally pooled together. Positive clones were finally selected against recombinant HA (first selection) and subsequently on HA gene transfected cells (second selection).

As evidenced by the reported examples, both B cells sources may potentially be useful but it is important to consider both the pros and the cons of each approach. In extreme synthesis, and as reported in Table 1, the choice of a proper B-cells donor and a proper B-cells source can be a crucial point for a successful cloning strategy. In particular, the source of B-cells must depend on the researcher’s purpose. In fact, when the main goal is the human administration of selected mAb, the choice of the B-cells donor should only be from humans. Otherwise, when possible administration in humans is not required (*i.e.*, such as in the case of development of diagnostic kits) or, when it is not possible to have access to human samples, the B-cells source can be the animal model.

#### 2.1.3. Libraries from Immunized Animals

Another approach to library design is its construction from B-cells obtained from immunized animals. This option is particularly useful especially when it is not possible to have access to human samples. Another potential advantage of immunized animals-derived libraries is the possibility of constructing them from animals immunized with less immunodominant, but potentially protective, antigenic regions that usually do not stimulate a strong response in humans. Those mentioned above are certainly the main advantages of using animal-derived libraries, but it is important to remember that this strategy is burdened by several drawbacks especially regarding the possible use of the cloned mAbs in humans. Non-human derived antibodies are generally burdened by side effects when used for human administration. For this reason, the use of non-human derived antibodies in clinical practice requires their previous “chimerization” (substitution of mouse constant regions with the homologous human regions) or “humanization” (reduction of mouse sequences only to the complementarity determining regions, CDRs), thus minimizing the non-human-derived mAb regions. Notwithstanding this, animal-derived mAbs may be very useful in the development of new diagnostic tools. An example of library construction from immunized animals used to develop new diagnostics is the work published by Goodchild *et al.*, describing the cloning of mAbs directed against the highly infectious Ebola virus [44]. In this work, the authors describe the molecular cloning of mAbs directed against Ebola virus to be used as immunodiagnostic reagents developed for in-field detection of Ebolavirus haemorrhagic fever. For the molecular cloning of such antibodies, two sharks (*Ginglymostoma cirratum*) were immunized with inactivated purified whole Zaire ebolavirus). Shark-derived Abs, as well as those obtained from camelids, are described as being thermo-stable when compared with human mAbs or scFv fragments. This is because camelids and sharks are known to possess functional homodimeric antibodies composed of only heavy chains in addition to classical heterodimeric immunoglobulin antibodies [19,20]. For this reason a phage display derived Ab selected from shark or camel B-cells is important by reason of its anti-Ebola use in remote environments on diagnostic grounds.

### 2.2. Panning Condition Optimization and Target Antigen Presentation as Key Points for Phage Display Success

Even if the library design and construction represents a crucial point, different panning strategies can be decisive for the molecular cloning of mAbs against infectious pathogens. The selection procedure utilizes the binding interactions between specific Abs presented on the bacteriophage and the target (generally coated in microtiter plates). The washing step follows the capturing step in order to wash away the unbound phages from the solid surface. Only the bound phages with strong affinity are kept. The final step involves the elution phase where the bound phages are eluted though changing the pH. The resulting filamentous phages can again infect the bacteria to improve the selection. The selection round can generally be repeated many times resulting in mAb strong affinity selection. Some examples of antigen presentation strategies are listed below and refer to the antibody molecular cloning against *Chlamydophila psittaci*, *Plasmodium falciparum* and HCV.

#### 2.2.1. Importance of the Selection Conditions

In this first example, several scFv-clones were selected capable of selectively recognizing *Chlamydophila psittaci*, discriminating it from the other chlamydiae. The aim of the study was to develop a diagnostic tool, and it was therefore easier to use B cells from immunized mice. The isolation of high affinity anti-carbohydrate scFv by phage display was performed in order to selectively recognize the *C. psittaci* carbohydrate antigens Kdo_4_ and 2.4/2.4Kdo_3_. The selection of anti-carbohydrate antigens antibodies was certainly the hardest task in this study. Once established the antigen presentation form, temperature and length of incubation were found to be the main parameters influencing the kinetic properties of phage display selected antibodies. For these reasons, the authors decided to focus the cloning strategy on the optimization of panning conditions, comparing two different selection conditions performed at two different temperatures [45].

The first condition consisted in performing panning at 4 °C and allowing a rapid isolation after three rounds of selection. After the first selection at 4 °C, none of the selected clones bound to immobilized *C. psittaci* in ELISA. In contrast, in the second approach, consisting of panning at 37 °C, the phage titer in ELISA against *C. psittaci* was noticeably increased. Moreover, it was empirically demonstrated that two additional rounds of selection performed at 4 °C resulted in further enrichment of phage binding to *C. psittaci*.

Temperature is not the only variable that can be modified when performing a selection procedure. Other features, such as the washing and elution conditions, the time of incubation and the correct antigen presentation format may be important, as evidenced by the following examples.

#### 2.2.2. Appropriate Selection of the Target Antigen and of the Correct Antigen Presentation

With this second example, we underline how both an appropriate antigen choice and presentation strategy, together with a good B-cell donor selection, can be effective for the cloning of mAbs directed against conserved motifs of hypervariable proteins. We present a selection strategy used for the molecular cloning of antibodies directed against the most important malaria surface protein (merozoite surface protein-1, MSP-1) [46]. Previous papers have demonstrated that Abs directed against MSP-1 are protective against *Plasmodium falciparum* in experimental animal infections [47,48]; moreover, the protein was successfully used as a protective immunogen in animal vaccine trials [49,50]. Most studies in fact, focus attention on the conserved C-terminal end of the protein (MSP-119) [51,52]. Not so much is known about the other MSP-1 regions, however there is a region (Block 2) representing the most variable domain present on this polymorphic protein. Considering block 2 sequence, it is possible to classify all MSP-1 variants into three different types (K1, MAD20 and RO33 types). An accurate study involving Block 2 structural characterization showed that two of the distinct protein types (K1 and MAD20) shared the presence of repeated structures, whereas the third type (RO33) contained a non-repetitive sequence well conserved between isolates. A positive correlation between protection against malaria and the presence of anti-Block 2 (B12) Abs has been demonstrated confirming that this region is important for the protection from *P. falciparum* infection [53–55].

Given this background, and after undertaking an accurate study on the malaria MSP-1 protein structure, a library from pooled peripheral blood lymphocytes belonging to ten patients with clinical symptoms of malaria was constructed. MAD20/B12 MSP-1, obtained from schizont-infected red blood cells and fused to glutathione-S-transferase (GST), was then used as the antigen for specific selection. Before the selection, the phage display library was depleted from GST-specific antibody fragments and then used for the selection of MSP-1 MAD20**/**Bl2-specific phage fragments. After three rounds of selection, Abs able to selectively bind to the antigen were obtained. The antigen presentation form selected by Sowa *et al.*, together with an accurate choice of B-cells donors, resulted in a good selection of mAbs able to bind a conserved region shared among *P. falciparum* isolates. More importantly, this successful cloning by phage display, represents an effective selective targeting example, basing its accomplishment on a deep antigen structural knowledge.

#### 2.2.3. Cross-Selection Strategies

As shown in the previous example, when coping with hypervariable pathogens, a really demanding but extremely interesting task is the isolation of mAbs directed against broadly shared conserved regions. The versatility of phage display could be of help. Below, we report two distinct examples of successful cross-selection strategies applied to a human viral pathogen endowed with high variability among its different genotypes: the hepatitis C virus.

HCV infects the human liver and it is estimated that 3% of humans carry a chronic infection. At this time, an effective anti-HCV vaccine is still not available and the therapeutic drugs used in clinical practice are burdened by considerable collateral effects [56–60]. The development of vaccines and drugs directed against the virus is impaired by the extreme HCV variability. In fact HCV genome replication is an error prone process and thus generates high genetic variability; as a consequence, within infected patients HCV is present as a cloud of distinct viruses (still genetically related) known as quasispecies.

All HCV isolates are actually clustered into seven genotypes. Each genotype shares two envelope proteins, E1 and E2, representing the most variable antigens of the virus. It is also demonstrated that Abs directed against E2, can neutralize the virus infectivity. Given the key role played by anti- HCV/E2 humoral immune response for its importance in inhibiting viral entry mechanisms and due to the extreme variability of HCV/E2, the chance to clone anti-HCV/E2 mAbs able to recognize more than a single HCV genotype is important for potential uses in HCV therapy.

The first report of human monoclonal recombinant HCV-specific Fabs obtained by Abs repertoire cloning in phage display combinatorial vectors was described by Burioni *et al.* [7,61]. An antibody phage combinatorial library was constructed from the bone marrow of a patient suffering from a chronic infection from HCV belonging to genotype 1b. The selection was performed [6,62] against HCV solid-phase bound HCV/E2 glycoprotein derived from a different genotype (genotype 1a), allowing the cloning of several Abs showing high affinity against the antigens [61]. In further studies the authors demonstrated that several clones exhibited a strong neutralizing activity against a multiplicity of HCV genotypes at very low concentrations [61,63–68]. This important result corroborates the importance of the right sample donor selection for the library construction. Moreover, it underlines the importance of the selection strategies adopted; in fact, the HCV antigens used for panning were of HCV genotype 1a. This shows how the cross-selection approach was effective for the selection of mAb able to recognize more than a single HCV genotype [7].

A second example of the key role of the panning strategy is the work conducted by Allander *et al*. [69] with the final goal of cloning anti-HCV mAbs. In this report the authors describe the isolation of anti-HCV Fab fragments derived, also in this case, from bone marrow-derived lymphocytes of a HCV infected patient. The experiment was designed to select anti-HCV/E2 cross-genotype reactive antibodies. Also in this case, the antigens used for the selection experiments were of different genotype (1a). As reported also by Burioni *et al.* [7], cross-genotyping selection can lead to the identification of antibodies directed against conserved epitopes. Allander *et al*. added several new panning strategies to the above mentioned approach. Indeed, panning was carried out essentially in three different panning series. In panning series I, three rounds of panning with recombinant E2 protein were performed increasing the number of washes for each round. The panning series II consisted of a single selection using the same antigen. Panning series III was performed as series I, but utilizing the recombinant HCV/E1E2 protein heterodimer. Also the second example successfully led to the cloning of different mAbs able to recognize and neutralize different HCV genotypes [70].

#### 2.2.4. Selection Using “Exhaustive Panning” Strategy

In a recent work published by Giang *et al*. [71], several human mAbs, recognizing distinct antigenic regions on the envelope glycoprotein complex E1E2 of HCV and endowed with broad neutralizing activity against diverse HCV genotypes, were selected from an HCV-immune phage-display Ab library by using an “exhaustive panning” strategy. In order to explore the antigenicity of E1E2 and select rare mAbs, an antibody library was screened repeatedly by using antigens masked with mAbs selected from the previous round [72,73]. This approach allowed the selection of mAbs with distinct binding properties at each new round of selection. The selection process was stopped only when no new mAb was recovered.

### 2.3. Phage Display for Crucial Post-Selection Optimization of mAbs

Phage display can be used for antibody library production and screening [74]. For applications related to therapeutic use, antibodies endowed with affinity in the lower nanomolar or subnanomolar range are often preferred [75]. For this reason, many antibody affinity maturation methods have been developed based on the introduction of mutations into the antibody genes in order to create a mutated antibody gene library. The mutagenesis can be performed by PCR site-specific mutation of complementarity determining regions (CDRs) [76]. Another strategy is random mutagenesis by the use of *E. coli* mutator strains or error-prone TempliPhi DNA amplification [77,78]. However, the most versatile technique is based on antibody gene amplification with an error-prone PCR [79,80]. In this case, only the genes of interest are mutated, permitting their subcloning into the phagemid in order to obtain a mini library containing different mutated variants of the original clone [75]. Below, some examples of mAbs optimization are described. From these phage display affinity maturation approaches, will result evidence that the success of the mAbs used in clinical practice (Palivizumab) as well as on clinical trial (Motavizumab, Anthim), can be specifically linked to the *in vitro* affinity maturation. It will be evident that the fastest and most successful way to improve the affinity still is the phage-display.

#### 2.3.1. Phage Display for mAb Optimization: The Example of Palivizumab and Motavizumab

The possibility of introducing post-cloning changes, allowed the introduction in clinical practice of a mAb directed against respiratory sinchythial virus (RSV) in human therapy. An example of mAb phage display optimization is represented by Palivizumab. It is well known that neutralizing antibodies play a fundamental role in protection against human RSV (hRSV) infection. The only RSV viral antigens capable of inducing neutralizing antibodies in animal models are F and G proteins [81,82]. The hRSV F protein is a type I glycoprotein that assembles as a homotrimer. Each monomer is synthesized as an inactive precursor (F0) that needs to be cleaved to acquire fusogenic activity. It has been postulated that the paramyxovirus F protein remains in a metastable prefusion conformation in the virus particle until the virus binds to the target. F protein is then activated to initiate conformational changes useful for the fusion process. After fusion, F acquires a highly stable conformation [83,84].

Palivizumab, an anti-respiratory syncytial virus (RSV) humanized monoclonal antibody (IgG1/κ), binds the hRSV F protein recognizing an epitope shared by both the hRSV F metastable prefusion form and the stable postfusion conformation [85]. The original antibody (mAb 1129) was derived from immunized mice [86]. The splenic lymphocytes of these mice were fused to a murine myeloma cell line to generate the 1129 hybridoma, which secreted an antibody neutralizing a broad spectrum of RSV isolates. The heavy and light chain variable domain genes from the mouse 1129 hybridoma were cloned and sequenced. Light chain CDRs of mAb 1129 were relocated onto the first three frameworks of a human light chain (K102) [87]. The fourth framework was derived from the human light chain gene Jκ 4 [88]. Similarly, heavy chain CDRs of mAb 1129 were transplanted into the first framework of a human heavy chain (Cor) [87] and the second, third, and fourth frameworks of another human heavy chain (CE-1) [87]. Palivizumab (MEDI-493) was generated by cloning this humanized Fv in frame with the constant regions of a human IgG1/κ immunoglobulin; Palivizumab heavy and light chain V region frameworks are then over 98% human. However, the unique characteristics of Palivizumab have been improved by an iterative approach of mutagenesis associated with phage display [89]. This crucial step in the setting up of Palivizumab was fundamental in the development of the molecule that was finally approved by FDA.

Since phage display engineering of Palivizumab has been successful in improving its activity, a further phage display optimization of Palivizumab was more recently performed leading to the development of a new mAb named Motavizumab. Motavizumab has both *in vitro* and *in vivo* improved potency compared to Palivizumab. The first step in engineering Palivizumab was to restore the parental LCDR1 sequence [90]. In addition, two murine residues in the Palivizumab sequence were mutated to human residues (both on the heavy and light chains). The engineered antibody, 493L1FR, has then a fully human framework sequence and similar binding affinity to F protein as Palivizumab and was used as a starting template for subsequent affinity maturation engineering [89].

In nature, high-affinity antibodies are generated by multiple rounds of somatic hypermutation followed by preferential B-cell clonal selection. Point mutations were generated in the CDR regions by an approach of oligonucleotide-based mutagenesis. Combinatorial libraries including these point mutations were constructed and then subjected to several rounds of affinity selection. Many point mutations were identified and several high-affinity combinatorial variants were selected [89]. These point mutations were located at four CDR positions. Affinity measurements of these variants in Fab format with BIAcore biosensor showed a three- to seven-fold increase in binding to F protein when compared with the original non-optimized Fab (493L1FR Fab). The best combinatorial Fab variants contained four mutations and exhibited a higher affinity than the Palivizumab Fab. The affinity improvement observed in these variants was feasible only when optimizing the mAb by phage display technology.

#### 2.3.2. Phage Display Optimization by Error-Prone Libraries Usage: The Example of Anthim

Here an example is reported of affinity maturation by phage display error-prone libraries usage. In this case, a monoclonal antibody directed against anthrax toxin was optimized.

The tripartite toxin produced by *Bacillus anthracis* is the principal determinant in the pathogenesis of anthrax. A panel of toxin-neutralizing antibodies was generated, including single-chain variable fragments (scFvs) and scFvs fused to a human constant κ domain (scAbs), characterized by the binding to the protective antigen (PA) subunit of the toxin [91]. For the generation of these high affinity mAbs directed against anthrax toxin, the experimental practice was mainly focused on phage display Ab-affinity maturation [91]. Both the heavy and light chain (HC and LC) variable regions derived from anti-PA hybridomas previously created [92,93] were cloned into an scFv phage display vector as described by Krebber *et al.* [94]. Monoclonal phage ELISA was used for PA-reactive clones identification. For antibody affinity maturation, libraries of mutated wild-type scFv positive clone were constructed using an error-prone PCR-based approach [95]. After DNA shuffling [96], the library was constructed in order to perform a screening of affinity-improved clones [91]. Panning was performed by ELISA wells with decreasing concentrations of PA, after blocking. Liquid-phase deselection of phage binding to PA with low affinity was performed on incubating phage and soluble PA. After washing, phage was used for a second round of panning. Each Ab-library was panned for five rounds before being screened for affinity-matured variants.

## 3. Conclusions

The availability of different phage display strategies can play an important role in the selection of mAbs targeting human pathogens. However, the final success of this extremely powerful technique depends on the choices made during the cloning process, starting from the selection of the right donor, through to the identification of the best antigen, and of the best selection conditions. It is important to remember that, even after a successful selection process, the features of a mAb maybe further improved in terms of affinity and neutralizing activity (Figure 1). In fact, several mAbs [97] are currently in clinical trials (Table 2) and it is easy to predict that very soon the only phage display derived mAb currently used in therapy (Palivizumab) will not be alone.

## Figures and Tables

**Table 1:** Advantages and disadvantages of alternative sources of phage-displayed antibody libraries.

Antibody library origin	PROs	CONs	Donor	B-cell source	PROs	CONs
**Humans**	Selection of mAbs potentially useful for human administration	Difficulties to obtain immunological reagents (*i.e.*, immunized humans)	Vaccinated donors/convalescent patients	Peripheral blood	Sample easy to obtain	Limited library extension
				Bone marrow	Library extension	Difficulties related to bone marrow sampling
**Animals**	Useful for diagnostic tools development or research usage; Possibility to immunize with synthetic molecules	Humanization or chimerization of selected mAbs required before human administration	Infected/immunized animals	Spleen/ Peripheral blood/Bone marrow	Easy sampling	
**Synthetic**	Library *in silico* design; No immunization or infection and tissue sampling required; Selective pathogen targeting	Possible limitations in library extension; Possible Ab misfolding and possible drawbacks for mAb production				

**Table 2:** Examples of clinical trials involving anti infectious monoclonal antibodies (mAbs); Light blue boxes highlight phage display-derived or optimized mAbs.

mAb Name	Origin	Ig Class or Ab Format	Molecular Target	Major Indication	Development Status
Edobacumab	*Mus Musculus*	IgM	Lipid A (LPS)	Septic Shock	Phase III
Nebacumab	*Homo sapiens*	IgM kappa	Lipid A (LPS)	Septic Shock	Phase I
Panobacumab	*Homo sapiens*	IgM kappa	*P. aeruginosa* serotype IATS O11	Nosocomial pneumonia caused by serotype 011 positive *P.aeruginosa*	Phase I
KB001	Human (from *Mus musculus*	Fab	*P. aeruginosa* PcrV	*P. aeruginosa* infection	Phase I/II
Felvizumab	Human (from *Mus musculus* )	IgG1	RSV Glycoprotein F	RSV infection	Phase III
Motavizumab (Numax^®^)	Human (from *Mus musculus* )	IgG1 kappa	RSV Glycoprotein F	RSV infection	Phase II
Palivizumab (Synagis^®^)	Human (from *Mus musculus* )	IgG1 kappa	RSV Glycoprotein F	RSV infection	Phase M
Sevirumab (Protovir™)	Human (from *Mus musculus* )	IgG1 kappa	HCMV gB glycoprotein gH envelope glycoprotein	HCMV infection	Phase III
Suvizumab	Human (from *Mus musculus* )	IgG1	HIV-1 IIIB gp120 V3 loop	HIV infection	Phase I
Tuvirumab	*Homo sapiens*			chronic HBV infection	Phase II
Efungumab (Mycograb®)	Phage display Human Antibody	Human scFv	Fungal HSP90	Fungal diseases	Phase III
Aurograb®	Phage display Human Antibody	Human scFv	Staph ABC transporter GrfA	MRSA, to be used with vancomycin	Phase III
Raxibacumab (Abthrax®)	Phage display Human Antibody	Human IgG	*B. anthracis* PA toxin	Anthrax biodefense	Phase III

## References

[1] Smith G.P. (1985). Filamentous fusion phage: Novel expression vectors that display cloned antigens on the virion surface. Science.

[2] Parmley S.F, Smith G.P. (1988). Antibody-selectable filamentous fd phage vectors: Affinity purification of target genes. Gene.

[3] Burioni R, Plaisant P, Delli Carri V, Vannini A, Spanu T, Clementi M, Fadda G, Varaldo P.E. (1997). An improved phage display vector for antibody repertoire cloning by construction of combinatorial libraries. Res. Virol.

[4] Mancini N, Carletti S, Perotti M, Canducci F, Mammarella M, Sampaolo M, Burioni R. (2004). Phage display for the production of human monoclonal antibodies against human pathogens. New Microbiol.

[5] Burton D.R, Barbas C.F., Persson M.A, Koenig S, Chanock R.M, Lerner R.A (1991). A large array of human monoclonal antibodies to type 1 human immunodeficiency virus from combinatorial libraries of asymptomatic seropositive individuals. Proc. Natl. Acad. Sci. USA.

[6] Williamson R.A, Burioni R, Sanna P.P, Partridge L.J, Barbas C.F., Burton D.R. (1993). Human monoclonal antibodies against a plethora of viral pathogens from single combinatorial libraries. Proc. Natl. Acad. Sci. USA.

[7] Burioni R, Plaisant P, Manzin A, Rosa D, Delli Carri V, Bugli F, Solforosi L, Abrignani S, Varaldo P.E, Fadda G (1998). Dissection of human humoral immune response against hepatitis C virus E2 glycoprotein by repertoire cloning and generation of recombinant Fab fragments. Hepatology.

[8] Zhang M.Y, Xiao X, Sidorov I.A, Choudhry V, Cham F, Zhang P.F, Bouma P, Zwick M, Choudhary A, Montefiori D.C (2004). Identification and characterization of a new cross-reactive human immunodeficiency virus type 1-neutralizing human monoclonal antibody. J Virol.

[9] Mao S, Gao C, Lo C.H, Wirsching P, Wong C.H, Janda K.D. (1999). Phage-display library selection of high-affinity human single-chain antibodies to tumor-associated carbohydrate antigens sialyl Lewisx and Lewisx. Proc. Natl. Acad. Sci. USA.

[10] Reiche N, Jung A, Brabletz T, Vater T, Kirchner T, Faller G. (2002). Generation and characterization of human monoclonal scFv antibodies against *Helicobacter pylori* antigens. Infect. Immun.

[11] Kim S.J, Jang M.H, Stapleton J.T, Yoon S.O, Kim K.S, Jeon E.S, Hong H.J. (2004). Neutralizing human monoclonal antibodies to hepatitis a virus recovered by phage display. Virology.

[12] Chang T.Y, Siegel D.L. (2001). Isolation of an IgG anti-B from a human Fab-phage display library. Transfusion.

[13] Roark J.H, Bussel J.B, Cines D.B, Siegel D.L. (2002). Genetic analysis of autoantibodies in idiopathic thrombocytopenic purpura reveals evidence of clonal expansion and somatic mutation. Blood.

[14] Clark M.A, Hawkins N.J, Papaioannou A, Fiddes R.J, Ward R.L. (1997). Isolation of human anti-c-erbB-2 Fabs from a lymph node-derived phage display library. Clin. Exp. Immunol.

[15] Roovers R.C, van der Linden E, de Bruine A.P, Arends J.W, Hoogenboom H.R. (2001). Identification of colon tumour-associated antigens by phage antibody selections on primary colorectal carcinoma. Eur. J. Cancer.

[16] Wu B.P, Xiao B, Wan T.M, Zhang Y.L, Zhang Z.S, Zhou D.Y, Lai Z.S, Gao C.F. (2001). Construction and selection of the natural immune Fab antibody phage display library from patients with colorectal cancer. World J. Gastroenterol.

[17] Xu M.Y, Xu X.H, Chen G.Z, Deng X.L, Li J, Yu X.J, Chen M.Z. (2004). Production of a human single-chain variable fragment antibody against esophageal carcinoma. World J. Gastroenterol.

[18] Sixholo J, van Wyngaardt W, Mashau C, Frischmuth J, Du Plessis D.H, Fehrsen J. (2011). Improving the characteristics of a mycobacterial 16 kDa-specific chicken scFv. Biologicals.

[19] De Genst E, Saerens D, Muyldermans S, Conrath K. (2006). Antibody repertoire development in camelids. Dev. Comp. Immunol.

[20] Dooley H, Flajnik M.F. (2006). Antibody repertoire development in cartilaginous fish. Dev. Comp. Immunol.

[21] Conrad U, Scheller J. (2005). Considerations on antibody-phage display methodology. Comb. Chem. High Throughput Screen.

[22] Chan C.E, Chan A.H, Lim A.P, Hanson B.J. (2011). Comparison of the efficiency of antibody selection from semi-synthetic scFv and non-immune Fab phage display libraries against protein targets for rapid development of diagnostic immunoassays. J. Immunol. Methods.

[23] Yu R, Wang S, Yu Y.Z, Du W.S, Yang F, Yu W.Y, Sun Z.W. (2009). Neutralizing antibodies of botulinum neurotoxin serotype a screened from a fully synthetic human antibody phage display library. J. Biomol. Screen.

[24] Tomlinson I.M, Cox J.P, Gherardi E, Lesk A.M, Chothia C. (1995). The structural repertoire of the human V kappa domain. EMBO J.

[25] Oliva B, Bates P.A, Querol E, Aviles F.X, Sternberg M.J. (1998). Automated classification of antibody complementarity determining region 3 of the heavy chain (H3) loops into canonical forms and its application to protein structure prediction. J. Mol. Biol.

[26] Clementi N, de Marco D, Mancini N, Solforosi L, Moreno G.J, Gubareva L.V, Mishin V, di Pietro A, Vicenzi E, Siccardi A.G (2011). PLoS One.

[27] Burioni R, Canducci F, Mancini N, Clementi N, Sassi M, de Marco D, Diotti R.A, Saita D, Sampaolo M, Sautto G (2010). Monoclonal antibodies isolated from human B cells neutralize a broad range of H1 subtype influenza A viruses including swine-origin Influenza virus (S-OIV). Virology.

[28] Burioni R, Canducci F, Mancini N, Clementi N, Sassi M, de Marco D, Saita D, Diotti R.A, Sautto G, Sampaolo M (2009). Molecular cloning of the first human monoclonal antibodies neutralizing with high potency swine-origin influenza A pandemic virus (S-OIV). New Microbiol.

[29] De Marco D.C.N, Mancini N, Solforosi L, Moreno G.J, Sun X, Tumpey T.M, Gubareva L.V, Mishin V, Clementi M, Burioni R. (2012). A non-VH1-69 heterosubtypic neutralizing human monoclonal antibody protects mice against H1N1 and H5N1 viruses. PLoS One.

[30] Burioni R, Mancini N, de Marco D, Clementi N, Perotti M, Nitti G, Sassi M, Canducci F, Shvela K, Bagnarelli P (2008). Anti-HIV-1 response elicited in rabbits by anti-idiotype monoclonal antibodies mimicking the CD4-binding site. PLoS One.

[31] Nabel G.J, Fauci A.S. (2010). Induction of unnatural immunity: Prospects for a broadly protective universal influenza vaccine. Nat. Med.

[32] Monto A.S, Ansaldi F, Aspinall R, McElhaney J.E, Montano L.F, Nichol K.L, Puig-Barbera J, Schmitt J, Stephenson I. (2009). Influenza control in the 21st century: Optimizing protection of older adults. Vaccine.

[33] Ansaldi F, Canepa P, Parodi V, Bacilieri S, Orsi A, Compagnino F, Icardi G, Durando P. (2009). Adjuvanted seasonal influenza vaccines and perpetual viral metamorphosis: The importance of cross-protection. Vaccine.

[34] Lambert L.C, Fauci A.S. (2010). Influenza vaccines for the future. N. Engl. J. Med.

[35] Stanekova Z, Vareckova E. (2010). Conserved epitopes of influenza A virus inducing protective immunity and their prospects for universal vaccine development. Virol. J.

[36] Steel J, Lowen A.C, Wang T.T, Yondola M, Gao Q, Haye K, Garcia-Sastre A, Palese P. (2010). Influenza virus vaccine based on the conserved hemagglutinin stalk domain. mBio.

[37] Desogus A, Burioni R, Ingianni A, Bugli F, Pompei R, Fadda G. (2003). Production and characterization of a human recombinant monoclonal Fab fragment specific for influenza A viruses. Clin. Diagn. Lab. Immunol.

[38] Karlsson Hedestam G.B, Fouchier R.A, Phogat S, Burton D.R, Sodroski J, Wyatt R.T. (2008). The challenges of eliciting neutralizing antibodies to HIV-1 and to influenza virus. Nat. Rev. Microbiol.

[39] Gamblin S.J, Skehel J.J. (2010). Influenza hemagglutinin and neuraminidase membrane glycoproteins. J. Biol. Chem.

[40] Mancini N, Solforosi L, Clementi N, de Marco D, Clementi M, Burioni R. (2011). A potential role for monoclonal antibodies in prophylactic. Antivir. Res.

[41] Kashyap A.K, Steel J, Rubrum A, Estelles A, Briante R, Ilyushina N.A, Xu L, Swale R.E, Faynboym A.M, Foreman P.K. (2010). Protection from the 2009 H1N1 pandemic influenza by an antibody from combinatorial survivor-based libraries. PLoS Pathog.

[42] Jayasekera J.P, Moseman E.A, Carroll M.C. (2007). Natural antibody and complement mediate neutralization of influenza virus in the absence of prior immunity. J. Virol.

[43] Throsby M, van den Brink E, Jongeneelen M, Poon L.L, Alard P, Cornelissen L, Bakker A, Cox F, van Deventer E, Guan Y (2008). Heterosubtypic neutralizing monoclonal antibodies cross-protective against H5N1 and H1N1 recovered from human IgM+ memory B cells. PLoS One.

[44] Goodchild S.A, Dooley H, Schoepp R.J, Flajnik M, Lonsdale S.G. (2011). Isolation and characterisation of Ebolavirus-specific recombinant antibody fragments from murine and shark immune libraries. Mol. Immunol.

[45] Gerstenbruch S, Brooks C.L, Kosma P, Brade L, Mackenzie C.R, Evans S.V, Brade H, Muller-Loennies S. (2010). Analysis of cross-reactive and specific anti-carbohydrate antibodies against lipopolysaccharide from *Chlamydophila psittaci*. Glycobiology.

[46] Sowa K.M, Cavanagh D.R, Creasey A.M, Raats J, McBride J, Sauerwein R, Roeffen W.F, Arnot D.E. (2001). Isolation of a monoclonal antibody from a malaria patient-derived phage display library recognising the Block 2 region of *Plasmodium falciparum* merozoite surface protein-1. Mol. Biochem. Parasitol.

[47] Knapp B, Hundt E, Enders B, Kupper H.A. (1992). Protection of Aotus monkeys from malaria infection by immunization with recombinant hybrid proteins. Infect. Immun.

[48] Perera K.L, Handunnetti S.M, Holm I, Longacre S, Mendis K. (1998). Baculovirus merozoite surface protein 1 *C*-terminal recombinant antigens are highly protective in a natural primate model for human Plasmodium vivax malaria. Infect. Immun.

[49] Diggs C.L, Ballou W.R, Miller L.H. (1993). The major merozoite surface protein as a malaria vaccine target. Parasitol. Today.

[50] Matsumoto S, Yukitake H, Kanbara H, Yamada T. (1999). Long-lasting protective immunity against rodent malaria parasite infection at the blood stage by recombinant BCG secreting merozoite surface protein-1. Vaccine.

[51] Burghaus P.A, Wellde B.T, Hall T, Richards R.L, Egan A.F, Riley E.M, Ballou W.R, Holder A.A. (1996). Immunization of Aotus nancymai with recombinant *C*-terminus of *Plasmodium falciparum* merozoite surface protein 1 in liposomes and alum adjuvant does not induce protection against a challenge infection. Infect. Immun.

[52] Chang S.P, Case S.E, Gosnell W.L, Hashimoto A, Kramer K.J, Tam L.Q, Hashiro C.Q, Nikaido C.M, Gibson H.L, Lee-Ng C.T (1996). A recombinant baculovirus 42-kilodalton C-terminal fragment of *Plasmodium falciparum* merozoite surface protein 1 protects Aotus monkeys against malaria. Infect. Immun.

[53] Cavanagh D.R, Elhassan I.M, Roper C, Robinson V.J, Giha H, Holder A.A, Hviid L, Theander T.G, Arnot D.E, McBride J.S. (1998). A longitudinal study of type-specific antibody responses to *Plasmodium falciparum* merozoite surface protein-1 in an area of unstable malaria in Sudan. J. Immunol.

[54] Miller L.H, Roberts T, Shahabuddin M, McCutchan T.F. (1993). Analysis of sequence diversity in the *Plasmodium falciparum* merozoite surface protein-1 (MSP-1). Mol. Biochem. Parasitol.

[55] Conway D.J, Cavanagh D.R, Tanabe K, Roper C, Mikes Z.S, Sakihama N, Bojang K.A, Oduola A.M, Kremsner P.G, Arnot D.E (2000). A principal target of human immunity to malaria identified by molecular population genetic and immunological analyses. Nat. Med.

[56] Choo Q.L, Kuo G, Weiner A.J, Overby L.R, Bradley D.W, Houghton M. (1989). Isolation of a cDNA clone derived from a blood-borne non-A, non-B viral hepatitis genome. Science.

[57] Arichi T, Saito T, Major M.E, Belyakov I.M, Shirai M, Engelhard V.H, Feinstone S.M, Berzofsky J.A. (2001). Retraction. Proc. Natl. Acad. Sci. USA.

[58] Weiner A.J, Brauer M.J, Rosenblatt J, Richman K.H, Tung J, Crawford K, Bonino F, Saracco G, Choo Q.L, Houghton M (1991). Variable and hypervariable domains are found in the regions of HCV corresponding to the flavivirus envelope and NS1 proteins and the pestivirus envelope glycoproteins. Virology.

[59] Bukh J, Purcell R.H, Miller R.H. (1993). At least 12 genotypes of hepatitis C virus predicted by sequence analysis of the putative E1 gene of isolates collected worldwide. Proc. Natl. Acad. Sci. USA.

[60] Prabhu R, Khalap N, Burioni R, Clementi M, Garry R.F, Dash S. (2004). Inhibition of hepatitis C virus nonstructural protein, helicase activity, and viral replication by a recombinant human antibody clone. Am. J. Pathol.

[61] Plaisant P, Burioni R, Manzin A, Solforosi L, Candela M, Gabrielli A, Fadda G, Clementi M. (1997). Human monoclonal recombinant Fabs specific for HCV antigens obtained by repertoire cloning in phage display combinatorial vectors. Res. Virol.

[62] Barbas C.F., Kang A.S., Lerner R.A., Benkovic S.J. (1991). Assembly of combinatorial antibody libraries on phage surfaces: The gene III site. Proc. Natl. Acad. Sci. USA.

[63] Mancini N, Diotti R.A, Perotti M, Sautto G, Clementi N, Nitti G, Patel A.H, Ball J.K, Clementi M, Burioni R. (2009). Hepatitis C virus (HCV) infection may elicit neutralizing antibodies targeting epitopes conserved in all viral genotypes. PLoS One.

[64] Perotti M, Ghidoli N, Altara R, Diotti R.A, Clementi N, de Marco D, Sassi M, Clementi M, Burioni R, Mancini N. (2008). Hepatitis C virus (HCV)-driven stimulation of subfamily-restricted natural IgM antibodies in mixed cryoglobulinemia. Autoimmun. Rev.

[65] Burioni R, Perotti M, Mancini N, Clementi M. (2008). Perspectives for the utilization of neutralizing human monoclonal antibodies as anti-HCV drugs. J. Hepatol.

[66] Mancini N, Carletti S, Perotti M, Romano L, Craxi R.D, Craxi A, Zanetti A.R, Clementi M, Burioni R. (2006). Modulation of epitope-specific anti-hepatitis C virus E2 (anti-HCV/E2) antibodies by anti-viral treatment. J. Med. Virol.

[67] Mancini N, Canducci F, Carletti S, Berardinelli E, Serafini G, Grieco A, Perotti M, Malcangi G, Danieli M.G, Varaldo P.E (2003). Heterogeneity of the humoral anti-HCV/E2 response in persistently infected patients as demonstrated by divergent patterns of inhibition of the binding of anti-HCV/E2 human monoclonal antibodies. J. Biol. Regul. Homeost. Agents.

[68] Burioni R, Matsuura Y, Mancini N, Tani H, Miyamura T, Varaldo P.E, Clementi M. (2002). Diverging effects of human recombinant anti-hepatitis C virus (HCV) antibody fragments derived from a single patient on the infectivity of a vesicular stomatitis virus/HCV pseudotype. J. Virol.

[69] Allander T, Drakenberg K, Beyene A, Rosa D, Abrignani S, Houghton M, Widell A, Grillner L, Persson M.A. (2000). Recombinant human monoclonal antibodies against different conformational epitopes of the E2 envelope glycoprotein of hepatitis C virus that inhibit its interaction with CD81. J. Gen. Virol.

[70] Johansson D.X, Voisset C, Tarr A.W, Aung M, Ball J.K, Dubuisson J, Persson M.A. (2007). Human combinatorial libraries yield rare antibodies that broadly neutralize hepatitis C virus. Proc. Natl. Acad. Sci. USA.

[71] Giang E, Dorner M, Prentoe J.C, Dreux M, Evans M.J, Bukh J, Rice C.M, Ploss A, Burton D.R, Law M. (2012). Human broadly neutralizing antibodies to the envelope glycoprotein complex of hepatitis C virus. Proc. Natl. Acad. Sci. USA.

[72] Ditzel H.J. (2002). Rescue of a broader range of antibody specificities using an epitope-masking strategy. Methods Mol. Biol.

[73] Tsui P, Tornetta M.A, Ames R.S, Silverman C, Porter T, Weston C, Griego S, Sweet R.W. (2002). Progressive epitope-blocked panning of a phage library for isolation of human RSV antibodies. J. Immunol. Methods.

[74] Sblattero D, Bradbury A. (2000). Exploiting recombination in single bacteria to make large phage antibody libraries. Nat. Biotechnol.

[75] Thie H, Voedisch B, Dubel S, Hust M, Schirrmann T. (2009). Affinity maturation by phage display. Methods Mol. Biol.

[76] Low N.M, Holliger P.H, Winter G. (1996). Mimicking somatic hypermutation: Affinity maturation of antibodies displayed on bacteriophage using a bacterial mutator strain. J. Mol. Biol.

[77] Irving R.A, Kortt A.A, Hudson P.J. (1996). Affinity maturation of recombinant antibodies using E. coli mutator cells. Immunotechnology.

[78] Fujii R, Kitaoka M, Hayashi K. (2004). One-step random mutagenesis by error-prone rolling circle amplification. Nucleic Acids Res.

[79] Martineau P. (2002). Error-prone polymerase chain reaction for modification of scFvs. Methods Mol. Biol.

[80] Tindall K.R, Kunkel T.A. (1988). Fidelity of DNA synthesis by the Thermus aquaticus DNA polymerase. Biochemistry.

[81] Stott E.J, Taylor G, Ball L.A, Anderson K, Young K.K, King A.M, Wertz G.W. (1987). Immune and histopathological responses in animals vaccinated with recombinant vaccinia viruses that express individual genes of human respiratory syncytial virus. J. Virol.

[82] Connors M, Collins P.L, Firestone C.Y, Murphy B.R. (1991). Respiratory syncytial virus (RSV) F, G, M2 (22K), and N proteins each induce resistance to RSV challenge, but resistance induced by M2 and N proteins is relatively short-lived. J. Virol.

[83] Lamb R.A. (1993). Paramyxovirus fusion: A hypothesis for changes. Virology.

[84] Lamb R.A, Jardetzky T.S. (2007). Structural basis of viral invasion: Lessons from paramyxovirus F. Curr. Opin. Struct. Biol.

[85] Magro M, Mas V, Chappell K, Vazquez M, Cano O, Luque D, Terron M.C, Melero J.A, Palomo C. (2012). Neutralizing antibodies against the preactive form of respiratory syncytial virus fusion protein offer unique possibilities for clinical intervention. Proc. Natl. Acad. Sci. USA.

[86] Beeler J.A, van Wyke Coelingh K. (1989). Neutralization epitopes of the F glycoprotein of respiratory syncytial virus: Effect of mutation upon fusion function. J. Virol.

[87] Kabat E.A, Wu T.T. (1991). Identical V region amino acid sequences and segments of sequences in antibodies of different specificities. Relative contributions of VH and VL genes, minigenes, and complementarity-determining regions to binding of antibody-combining sites. J. Immunol.

[88] Hieter P.A, Maizel J.V., Leder P. (1982). Evolution of human immunoglobulin kappa J region genes. J. Biol. Chem.

[89] Wu H, Pfarr D.S, Tang Y, An L.L, Patel N.K, Watkins J.D, Huse W.D, Kiener P.A, Young J.F. (2005). Ultra-potent antibodies against respiratory syncytial virus: Effects of binding kinetics and binding valence on viral neutralization. J. Mol. Biol.

[90] Johnson S, Oliver C, Prince G.A, Hemming V.G, Pfarr D.S, Wang S.C, Dormitzer M, O’Grady J, Koenig S, Tamura J.K (1997). Development of a humanized monoclonal antibody (MEDI-493) with potent *in vitro* and *in vivo* activity against respiratory syncytial virus. J. Infect. Dis..

[91] Maynard J.A, Maassen C.B, Leppla S.H, Brasky K, Patterson J.L, Iverson B.L, Georgiou G. (2002). Protection against anthrax toxin by recombinant antibody fragments correlates with antigen affinity. Nat. Biotechnol.

[92] Little S.F, Novak J.M, Lowe J.R, Leppla S.H, Singh Y, Klimpel K.R, Lidgerding B.C, Friedlander A.M. (1996). Characterization of lethal factor binding and cell receptor binding domains of protective antigen of *Bacillus anthracis* using monoclonal antibodies. Microbiology.

[93] Little S.F, Leppla S.H, Cora E. (1988). Production and characterization of monoclonal antibodies to the protective antigen component of *Bacillus anthracis* toxin. Infect. Immun.

[94] Krebber A, Bornhauser S, Burmester J, Honegger A, Willuda J, Bosshard H.R, Pluckthun A. (1997). Reliable cloning of functional antibody variable domains from hybridomas and spleen cell repertoires employing a reengineered phage display system. J. Immunol. Methods.

[95] Fromant M, Blanquet S, Plateau P. (1995). Direct random mutagenesis of gene-sized DNA fragments using polymerase chain reaction. Anal. Biochem.

[96] Stemmer W.P. (1994). Rapid evolution of a protein *in vitro* by DNA shuffling. Nature.

[97] Ter Meulen J. (2011). Monoclonal antibodies in infectious diseases: Clinical pipeline in 2011. Infect. Dis. Clin. N. Am.

